# A Visual Pathway Links Brain Structures Active during Magnetic Compass Orientation in Migratory Birds

**DOI:** 10.1371/journal.pone.0000937

**Published:** 2007-09-26

**Authors:** Dominik Heyers, Martina Manns, Harald Luksch, Onur Güntürkün, Henrik Mouritsen

**Affiliations:** 1 AG Neurosensorik, Institute of Biology, University of Oldenburg, Oldenburg, Germany; 2 Department of Biopsychology, Institute for Cognitive Neuroscience, Ruhr-University Bochum, Bochum, Germany; 3 Chair of Zoology, Department of Zoology, Technical University Munich, Freising-Weihenstephan, Germany; University of Alberta, Canada

## Abstract

The magnetic compass of migratory birds has been suggested to be light-dependent. Retinal cryptochrome-expressing neurons and a forebrain region, “Cluster N”, show high neuronal activity when night-migratory songbirds perform magnetic compass orientation. By combining neuronal tracing with behavioral experiments leading to sensory-driven gene expression of the neuronal activity marker ZENK during magnetic compass orientation, we demonstrate a functional neuronal connection between the retinal neurons and Cluster N via the visual thalamus. Thus, the two areas of the central nervous system being most active during magnetic compass orientation are part of an ascending visual processing stream, the thalamofugal pathway. Furthermore, Cluster N seems to be a specialized part of the visual wulst. These findings strongly support the hypothesis that migratory birds use their visual system to perceive the reference compass direction of the geomagnetic field and that migratory birds “see” the reference compass direction provided by the geomagnetic field.

## Introduction

The navigational abilities of birds have fascinated mankind for centuries and challenged researchers for decades. Behavioral experiments have shown that night-migratory passerine birds can use a magnetic compass to orient during migration [e.g. 1], [2] and recent data suggest that the magnetic compass is used as the birds' primary compass in mid-air during real migratory flights [3]. Nevertheless, the neuronal mechanisms underlying their magnetosensory abilities remain elusive.

Currently, theoretical, behavioral and physiological evidences support two magnetic sensing hypotheses: a magnetite-mediated magnetic sense [4]–[6] and/or a vision-mediated magnetic compass [7]. The magnetite-mediated mechanism seems to act as part of a magnetic map- or signpost sense, which could provide the animal with information about its geographic position, whereas the vision-mediated magnetic sense seems to be a pure compass sense that is based on radical-pair processes in the birds' eye(s) [for reviews], [see e.g. 8, 9]. The light-dependent, radical-pair based, magnetic compass hypothesis suggests that magnetic modulations of radical-pair processes in photoreceptor molecules in the birds' eyes provide information about the individual's orientation relative to the magnetic field lines [7], [10].

Putative sensor molecules (cryptochromes) which seem to possess the required biophysical characteristics, have recently been shown to be expressed in the retina of migratory birds [11], [12]. In garden warblers, *Sylvia borin*, the cryptochrome-expressing retinal ganglion cells and a neuronal cluster located in posterolateral regions of both forebrain hemispheres (“Cluster N”) show high, sensory-driven neuronal activity as indicated by the expression of the Immediate Early Gene ZENK during magnetic orientation [12]–[14]. Strong neuronal activation in Cluster N is only observed at night in migratory birds but not in non-migratory zebra finches, and the activation in the migrants is absent when the birds' eyes are covered, suggesting that some kind of night vision specialization in night-migratory birds is involved in activation of Cluster N. We have suggested [13] that night migratory birds may use Cluster N for seeing better at night and/or for visual night-time navigation. We furthermore suggested that Cluster N is likely to process such light-mediated magnetic compass responses, based on the fact that Cluster N is the only known forebrain area that is highly active during magnetic compass orientation, and on the theoretical model [7] on magnetic field modulation of the light sensitivity of specialized receptor molecules in the retina of the birds, [for detailed arguments see 13], [14].

Sensory systems process their particular stimuli in specific brain circuits and pathways. Thus, the identification of what sensory system(s) is active during magnetic compass orientation, provides a way to recognize the sensory quality utilized during that specific behavior. Therefore, the aim of the present study was to investigate whether and if so, how Cluster N and the retina are interconnected. To do this, we combined neuronal tracing with analyses of ZENK expression as a marker for neuronal activity induced during behavioral experiments.

## Results

By tracing retinal projections to the brain and simultaneously labeling connections innervating Cluster N, we found colocalization of the tracers in specific substructures of the visual thalamus.

**Figure 1:**
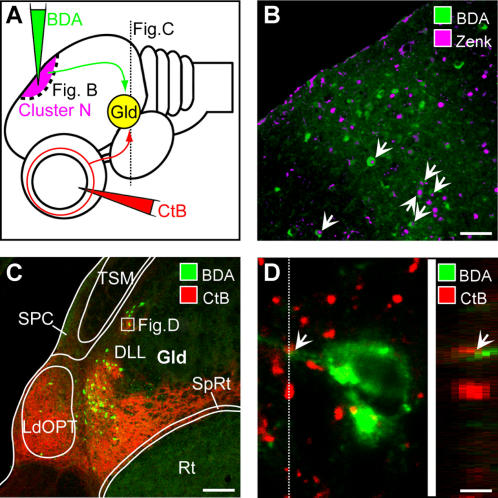
Neuronal tracing reveals that Cluster N receives input through the thalamofugal visual pathway. A: Schematic side view of the bird's brain indicating the locations of tracer application. Retrograde tracer (BDA, shown in green) was iontophoretically applied into Cluster N (shown in magenta). Anterograde tracer (CtB, shown in red) was injected into the vitreous of the contralateral eye. B: Double-labeling of ZENK and the retrograde tracer BDA in sagittal brain sections at the level of Cluster N proves the correct placement of tracer into Cluster N: arrows point to examples of neurons displaying ZENK-immunoreactivity (shown in magenta) in the nucleus together with BDA (shown in green) in the somata. Scale bar: 25 µm. C: Tracer distribution in frontal brain sections at the level of the thalamic Gld. Anterogradely labeled fibers from the retina (shown in red) project upon all substructures of the Gld, i.e. LdOPT, SpRt and lateral/ventral parts of the DLL. Retrogradely labeled neurons projecting upon Cluster N (visualised green) mainly originate within the DLL, with few additional connections from the LdOPT and SpRt. Scale bar: 50 µm. D: Confocal 3D-stacks in the thalamic Gld at high magnification indicate direct contact (arrows) between retinofugal fibers (shown in red) and somata/proximal dendrites retrogradely labeled from Cluster N (shown in green). Scale bar: 4 µm. Abbreviations: DLL, Nucleus dorsolateralis anterior thalami, pars lateralis; Gld, dorsolateral geniculate complex; LdOPT, Nucleus lateralis dorsalis nuclei optici principalis thalami; Rt, Nucleus rotundus; SPC, Nervus superficialis parvocellularis; SpRt, Nucleus suprarotundus; TSM, Tractus septomesencephalicus.

**Figure 2:**
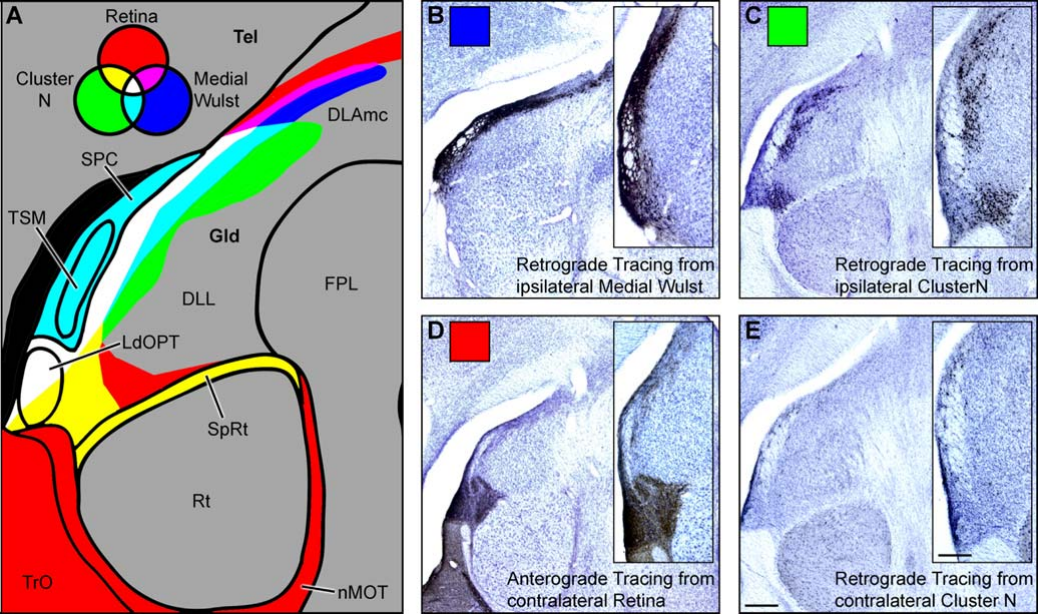
Cluster N is linked to a specific subsystem of the thalamofugal visual pathway. A: Schematic overview of anatomical features according to cresylviolet staining (shown in B, C and D) and tracing patterns in the thalamus. Each input source (retina, red; medial wulst, blue; Cluster N, green) and their overlapping tracing patterns are color-coded separately. Retinofugal fibers show a high degree of overlap with neurons projecting upon Cluster N (shown in yellow) in ventral parts of the DLL, the SpRt and the LdOPT, whereas neurons projecting upon the medial wulst exclusively co-localize with retinofugal fibers only in dorsal DLL regions and the DLAmc (shown in magenta). Lateral parts of the DLL, which receive afferents from the retina, project upon the medial wulst and Cluster N (shown in white). B: Retrograde tracing pattern from the medial visual wulst, ipsilateral side. Neurons are mainly located in lateral parts of the DLL and LdOPT with few single neurons positioned in lateral parts of the DLAmc. C: Retrograde tracing pattern from Cluster N, ipsilateral side. Labeled neurons cover lateral and ventral parts of the DLL and are found in the SpRT and LdOPT (compare Fig. 1C). D: Contralaterally projecting retinofugal fibers innervate the LdOPT, SpRt and lateral/ventral parts of the DLL, the nMOT and a small band along the lateral DLL reaching parts of the DLAmc (compare Fig. 1C). E: Retrograde tracing pattern from Cluster N, contralateral side. Few scattered neurons are found in DLL and LdOPT. Scale bar (for B–E): 100 µm; scale bar in insert (for insert in B–E): 50 µm. Abbreviations: AL, Ansa lenticularis; DLAmc, Nucleus dorsolateralis anterior thalami, pars magnocellularis: DLL, Nucleus dorsolateralis anterior thalami, pars lateralis; FPL, Fasciculus prosencephali lateralis; LdOPT, Nucleus lateralis dorsalis nuclei optici principalis thalami; nMOT, Nucleus marginalis tractus optici; OM, Tractus occipitomesencephalicus; Rt, Nucleus rotundus; SPC, Nervus superficialis parvocellularis; SpRt, nucleus suprarotundus; Tel, Telencephalon; TrO, Tractus opticus; TSM, Tractus septomesencephalicus.

The anterograde (forward) tracing results of retinal projections in the garden warbler demonstrated virtually identical connections between the eye and the brain as known from other lateral-eyed bird species [e.g. 15]–[18]: fibers either projected onto the contralateral optic tectum (part of the tectofugal system), to the nucleus of the basal optic root (part of the accessory pathway) (data not shown) or curved into the thalamus innervating the dorsal geniculate complex (Gld, for details, see Fig. 1C, 2A, D).

Tracer applied into Cluster N labeled neuronal somata in the Gld regions of both hemispheres with the vast majority being located on the ipsilateral side relative to Cluster N. On the ipsilateral side, the neurons projecting onto Cluster N (Fig 1C, 2A, shown in green; Fig. 2C, shown in black) were mainly located in lateral and ventral parts of the DLL (Nucleus dorsolateralis anterior thalami, pars lateralis) with few additional connections from the LdOPT (Nucleus lateralis dorsalis nuclei optici principalis thalami) and SpRt (Nucleus suprarotundus). The distribution of the few retrogradely labeled neurons in the contralateral Gld mirrored the results seen on the ipsilateral side (Fig. 2E).

**Figure 3:**
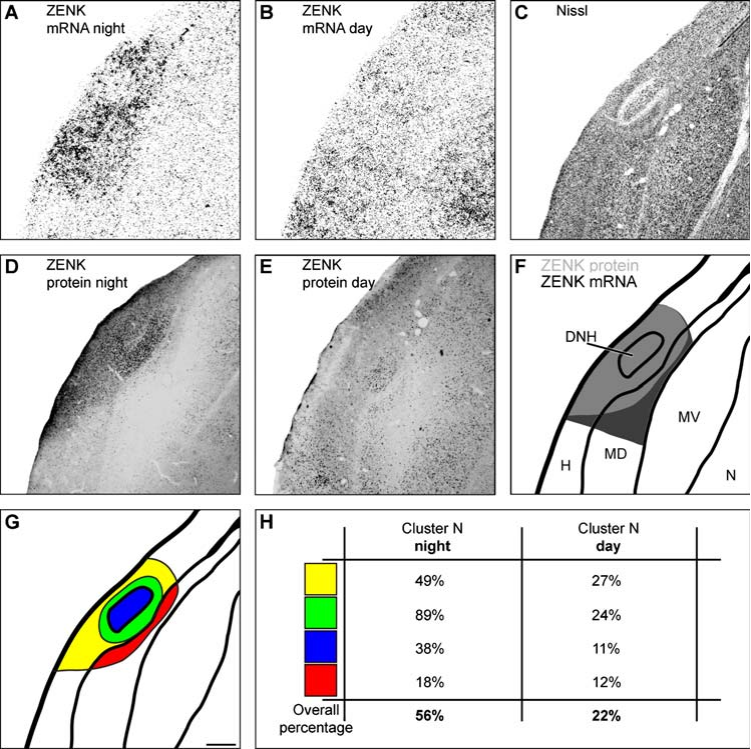
Detailed quantification of ZENK protein expression and comparison with ZENK mRNA expression within Cluster N. A: Expression of ZENK mRNA during night-time covers posterolateral parts of the hyperpallium and underlying mesopallium. In the DNH nucleus, the amount of ZENK mRNA transcripts is decreased. D: Expression of ZENK protein during night-time covers hyperpallial compartments comparable to the expression of ZENK mRNA but decreases in mesopallial portions. Within Cluster N, approximately 56% of neurons show nuclear expression of ZENK protein with highest relative amounts of ZENK-positive nuclei found in the shell surrounding the DNH nucleus. B: Decreased expression of ZENK mRNA and E: protein during day in the whole hyperpallium. Nuclear ZENK protein is found in approximately 22% of Cluster N neurons. Note that, ventral mesopallial (MV) and nidopallial (N) portions show increased ZENK expression on the mRNA and protein level compared to night-time activation patterns. C: Corresponding Nissl-stained section and F: schematic drawing display morphological features and neuroanatomical location of Cluster N within the telencephalon. G: Determination of four subregions within Cluster N defined by morphological boundaries (compare Fig. 3C): DNH nucleus (Fig. 3G, shown in blue); the shell surrounding DNH nucleus (Fig. 3G, shown in green); the remaining hyperpallial Cluster N part (Fig. 3G, shown in yellow); the mesopallial Cluster N part (Fig. 3G, shown in red). Scale bar (for A–G): 250 µm. H: Quantification of percentages of neurons with nuclear expression of ZENK within each subunit. Abbreviations: DNH, dorsal nucleus of the hyperpallium; H, hyperpallium, MD, dorsal mesopallium; MV, ventral hyperpallium; N, nidopallium.

The proper placement of retrograde tracer into Cluster N was proven by co-localization of tracer and the neuronal activity marker ZENK, since Cluster N is the only part of the forebrain displaying movement-independent ZENK activity in night-migratory birds sitting still or performing magnetic compass orientation under dim light conditions at night [13]. A total of 21 birds have been analyzed in this study. For all cases, no principally different individual variation in ZENK expression within Cluster N has been observed. When tracer was applied into Cluster N, double labeling showed nuclear expression of ZENK (Fig. 1B, shown in pink), while the injected retrograde tracer was found in somatic cell compartments (Fig. 1B, shown in green). ZENK protein immunoreactivity displayed a similar pattern as previously shown for the expression of ZENK mRNA [Fig. 3; (13)]. Slight differences were observed in mesopallial parts of Cluster N (MD in Fig. 3F). Relative amounts of ZENK expressing neurons (detailed values shown in Fig. 3H) were determined for four subunits within Cluster N, each of them separated by clearly visible morphological boundaries (compare Fig. 3C): first, the DNH nucleus (Fig. 3G, shown in blue); second, the shell surrounding the DNH nucleus, characterized by small, densely packed neurons (Fig. 3G, shown in green); third, the remaining hyperpallial part of Cluster N (Fig. 3G, shown in yellow); fourth, the mesopallial part of Cluster N (Fig. 3G, shown in red). In total, approximately 56% of all Cluster N neurons show nuclear expression of ZENK protein at night (Fig. 3D, H). The highest density of ZENK-positive neurons was found in the shell surrounding the DNH nucleus (89%; Fig. 3G/H, shown in green). In comparison, few ZENK expressing neurons within all subunits of Cluster N are observed during daytime. The overall percentage of ZENK-expressing neurons within Cluster N drops to approximately 22% during the day (Fig. 3E, for detailed values, see Fig. 3H).

Co-localization of anterograde tracer from the eye and retrograde tracer from Cluster N was observed in lateral and ventral parts of the DLL, the LdOPT and the SpRt of the thalamic Gld. Confocal stacks at high magnification of these Gld regions revealed a close vicinity between retinofugal fiber endings (Fig. 1D, shown in red) and somata/proximal dendrites of neurons projecting upon Cluster N (Fig. 1D, shown in green). Although there may also be further connections involving interneurons, the thalamic Gld, in many bird species, shows a strictly focused projection upon the visual wulst. Therefore, these data strongly suggest that some retinal ganglion cells innervate neurons that project upon Cluster N (arrows in Fig. 1D).

To differentiate Cluster N connectivity from those of the rest of the visual wulst, three birds underwent tracer application into wulst areas medial to Cluster N at the same rostro-caudal level. Sections were counter-stained with cresyl-violet for neuroanatomical orientation. In all three cases, retrogradely labeled neurons were located along a thin band covering dorsal and lateral DLL parts of the thalamic Gld reaching the dorsomedially located DLAmc (Nucleus dorsolateralis anterior thalami, pars magnocellularis; Fig. 2A, shown in blue, B, shown in black). By comparing the retrograde tracing patterns from Cluster N to those from the medial wulst, lateral-most parts of the DLL were shown to innervate both the medial and lateral visual wulst regions and to co-localize with fibers anterogradely traced from the retina (Fig. 2A, shown in white). In contrast, the DLAmc and dorsal parts of the DLL exclusively showed overlap between tracer from the retina and neurons innervating the medial visual wulst (Fig. 2A, shown in magenta), whereas overlap between retinofugal fibers and neurons projecting only upon Cluster N was observed in ventral parts of the DLL and parts of the LdOPT and SpRt (Fig. 2A, shown in yellow).

## Discussion

The present data demonstrate an anatomical connection between the retina and Cluster N through the thalamus. This shows that Cluster N receives sensory input from the eyes and suggests that Cluster N is at least partly located in the visual Wulst. In general, the Wulst is the telencephalic termination area of the thalamofugal pathway which conveys visual input from the retina onto the forebrain via the Gld [18], [19]. We show here with retrograde tracing that Cluster N receives input from the Gld suggesting that at least parts of Cluster N belong to the visual wulst. More specifically, our focal tracer injections revealed that Cluster N is connected to a specific subsystem of the thalamofugal pathway. We could demonstrate that the projections from the thalamus upon the visual wulst in the garden warbler are organized in a topographic fashion as it was also shown in pigeons [20] and chicks [21]. This organization indicates that the thalamofugal system is structured into parallel, functionally segregated pathways which may process different aspects of visual stimuli [21], comparable to what is known from the tectofugal pathway [22], [23]. While Cluster N is innervated by latero-ventral Gld nuclei, portions of the visual wulst located medial from Cluster N receive input from dorso-medial thalamic neurons. These neuronal populations did not show as much overlap with retinal fibers as the ones projecting upon Cluster N. Thus, Cluster N is primarily connected to the thalamofugal subsystem that receives strong retinal input. This finding implies that visual information is the major input to Cluster N and supports the idea that magnetic compass orientation is linked to night vision.

The vast majority of forebrain neurons in songbirds can express ZENK as a marker for neuronal activity [13], [ 14], [ 24]–[26] Feenders, Liedvogel, Zapka, Mouritsen, Jarvis, personal communication]. Furthermore, movement-independent ZENK expression in the forebrain of night-migratory birds performing magnetic orientation at night is confined to Cluster N [with the strongest activation in distinct subregions (the shell surrounding the DNH nucleus)], as shown by the detailed quantification of ZENK expression within Cluster N performed in this study], and this expression massively decreased in corresponding brain areas of non-migratory songbirds and in all bird species during daytime [13]. Seen together, these findings strongly support the suggestion that Cluster N processes some kind of night-time visual information processing which is a specialization of night-migratory birds [for detailed arguments], [see 13, 14]. Combined with these findings, the present tracing data for the first time suggest a putative magnetosensory compass pathway from the sensory organ (the eye) to its main integrative brain center (Cluster N) in night-migratory birds. This putative compass-magnetosensory pathway involves restricted subregions at all levels of the thalamofugal visual pathway: neuronal subpopulations in the retina, ventral parts of the thalamic Gld (lateral and ventral DLL, SpRt, LdOPT) and lateral parts of the visual wulst (Cluster N). Due to the fact that a known visual pathway connects the only brain structures that have been shown to be active during magnetic orientation, our findings strongly support the hypothesis that migratory birds perceive the magnetic field as a visual pattern and that they are thus likely to “see” the magnetic field.

## Materials and Methods

### Animals and housing conditions

All animal procedures were approved by the local and national authorities for the use of animals in research. Adult garden warblers (*Sylvia borin*) were obtained from bird banding stations in Helgoland (Germany) and Rybachy (Russia). The birds were housed in single wire cages and experienced a circadian and circannual light regime closely matching the natural conditions in Oldenburg, Germany. All birds got used to captivity for at least 2 weeks before getting involved in any experiment. Food and water were provided ad libitum.

### Axonal Tracing

A total of 21 birds were used in this study (see table 1). For tracer injections, birds were anaesthetized by intramuscular injection of ketamine (Pfitzer, Freiburg, Germany)/rompun (Bayer, Leverkusen, Germany), and their heads were fixed in a custom-built stereotactic apparatus. The skin on the birds' head was anaesthetized using a surface anaesthetic (Xylocain; Astra Zeneca, Wedel, Germany) and incised dorsally. For tracer injections into the visual Wulst, application coordinates were determined relative to the prominent bifurcation of the Y-sinus [27] as an initial reference. A small part of the skull was carefully removed above the respective brain region. Afferents to the visual wulst were mapped by stereotactic iontophoretic application (4 µA positive current, 7 sec on/off, duration: 20–30 min) of biotinylated dextran amin (BDA, working dilution: 10% in phosphate buffered saline, PBS; Molecular Probes Europe BV, Leiden, The Netherlands) into Cluster N or into medial parts of the visual Wulst. After the surgery, the skin on the bird's head was re-sealed with cyanoacrylate surgical glue (Glubran, Viareggio, Italy). Afterwards, anterograde connections from the retina were mapped by microinjection of 5 µl Choleratoxin B subunit (CtB, working dilution: 1% in distilled water; Sigma, Deissenhofen, Germany) in the vitreous of the eye contralateral to the Wulst injection.

**Table 1:** Overview of tracer application sites and number of specimens used.

Treatment of specimen	Number of specimens
Anterograde tracing from the eye only	2
Retrograde tracing from the lateral visual wulst = Cluster N only	7
Anterograde tracing from the eye and retrograde tracing from the lateral visual wulst = Cluster N	9
Retrograde tracing from the medial visual wulst only	3
**Total number of specimens used:**	**21**

### Behavioral analysis

Forty-eight to seventy-two hours after surgery, single garden warblers were put into a custom-built, cylindrical plexiglass cage fitted with a circular perch in the center [28]. To allow acclimatization to the new surroundings, birds were placed in the cages at least 2 hours before the experiment started. At dusk, room lights were reduced to a light intensity of 0,04 lux, a typically used value for behavioral orientation tests using night migrants [e.g. 1], [ 13], [ 29]–[31]. To minimize brain activity evoked through any sensory or motoric disturbances, we only collected birds after they had been sitting relatively still and constantly awake for at least 2 hours in the cage under the low light conditions. “Relatively still” means that the birds did perform head scans [28] and did occasionally move around on the perch in all cases together with minimal (<5 mins/h) unspecific motor activity (flying around/jumping on/off the perch, as this would have led to motor-dependent activity in the brain). Each bird's behavior was continuously observed in real-time by the experimenter with an infrared camera (840 nm) connected to a surveillance monitor to make sure that the bird is awake (eyes open) as this is a prerequisite for Cluster N activation [13].

### Processing of brain tissue

At the end of the experiment, birds were killed by an overdose of Narcoren (Merial, Hallbergmoos, Germany) and transcardially perfused with 0,12 M phosphate buffered saline (PBS) followed by 4% paraformaldehyde (PFA) dissolved in PBS. The brains were dissected from the skull and post-fixed in 4% PFA dissolved in PBS for 3 hours. Tissue was cryoprotected in 30% Sucrose dissolved in PBS for 24 h and cut on a freezing microtome (Leica 1850, Solms, Germany) in six series of 40 µm thick sections in either the frontal or the sagittal plane. Sections were stored in PBS containing 0,01% Na-azide at 4°C.

### Immunohistochemical stainings

Brain slices were reacted free-floating according to the immuno-ABC-technique [32]. Each incubation step was followed by rinsing sections three times in PBS for 5 minutes each. Endogenous peroxidases were inactivated by incubation with 0,3% hydrogen peroxide dissolved in distilled water for 60 minutes and unspecific binding sites were blocked by incubating the slices in 10% normal serum dissolved in PBS containing 0,3% Triton-X100 (PBS-T, Sigma, Deissenhofen, Germany) or in 10% fetal calf serum (Kraeber, Ellerbek, Germany) for 60 minutes. Slices were incubated with the primary antibody for 3 days (polyclonal rabbit raised against Egr-1/ZENK (Santa Cruz, CA), 1∶1000; polyclonal rabbit raised against CtB (Sigma, Deisenhofen, Germany), 1∶500 in PBS-T). Thereafter, sections were sequentially incubated for 60 minutes each with biotinylated secondary antibodies and avidin-coupled peroxidase-complex (Vector ABC Elite Kit, Vector Laboratories, Burlingame, CA). After washing, the peroxidase-activity was detected using a 3′3-diaminobenzidine (DAB; Sigma, Deisenhofen, Germany) reaction, modified by the use of β-d-glucose/ glucose-oxidase (Sigma, Deisenhofen, Germany; 33). The reaction was stopped by transferring the sections into PBS. Sections were mounted on gelatinized slides, dehydrated, and embedded in Entellan (Merck, Darmstadt, Germany).

For colocalization of both tracers or one tracer together with ZENK signals, primary antibodies (Egr-1/ZENK, 1∶500; CtB, 1∶300 in PBS-T) were detected by an appropriate secondary antibody (polyclonal goat raised against rabbit IgG labeled with fluorescent dyes Alexa488; Molecular Probes Europe BV, Leiden, The Netherlands, 1∶400 in PBS-T). BDA was detected by streptavidine labeled with fluorescent dye Alexa 555 (Molecular Probes Europe BV, Leiden, The Netherlands, 1∶400 in PBS-T). Sections were mounted on gelatinized glass slides and coverslipped with Vectashield medium (Vector Laboratories, Burlinghame, CA).

### Analysis, digital processing and photomicrograph production

Sections at all levels of the brain were analyzed. Depending on the staining procedure, either a stereomicroscope (Leica M, Leica IM 50, Solms, Germany) or a confocal microscope (Leica DMR-E, Nussloch, Germany) was used for documentation of representative digital images shown in this article. Schematic drawings, labeling and layout were done using the Photoshop 6.0 and Illustrator 10.0 software (Adobe Systems, Mountain View, CA). Neuroanatomical structures were named by using brain atlases of chicken [34], pigeon [35] and canary [27]. Quantification of relative amounts of neurons expressing ZENK was done by estimating the total number of neurons on Nissl-stained sections in defined areas of Cluster N. In corresponding sections immunolabeled against ZENK protein, ZENK-positive nuclei were counted.
